# A Lysis- and Sonication-Based Method for the Quantification of Extracellular Vesicle-Bound Cardiac Troponin T Using a High-Sensitivity Immunoassay

**DOI:** 10.3390/biomedicines14081653

**Published:** 2026-07-23

**Authors:** Yuetong Leona Ding, Dominika Bernath-Nagy, Chiara Heß, Florian Leuschner, Hugo Albert Katus, Norbert Frey, Jona Benjamin Krohn, Evangelos Giannitsis

**Affiliations:** 1 Department of Cardiology, Pulmonology and Angiology, University Hospital Heidelberg, 69120 Heidelberg, Germany; 2 Heart and Vascular Centre, Semmelweis University, 1122 Budapest, Hungary; 3 German Centre for Cardiovascular Research (DZHK), Partner Site Heidelberg/Mannheim, 69120 Heidelberg, Germany

**Keywords:** extracellular vesicles, cardiac troponin T, hs-cTnT assay, myocardial injury, myocardial ischemia, sonication, troponin compartmentation, cardiac biomarker

## Abstract

**Background/Objectives:** High-sensitivity cardiac troponin (hs-cTn) assays are used in routine diagnostics to detect myocardial injury. However, a fraction of circulating cardiac troponin T (cTnT) enclosed within extracellular vesicles (EVs) goes widely undetected. This study introduces a combined lysis- and sonication-based protocol to release and quantify EV-bound cTnT in a time-efficient manner using a state-of-the-art hs-cTnT immunoassay. **Methods:** Plasma samples from patients with non-ST-segment elevation myocardial infarction (NSTEMI), unstable angina, pulmonary embolism, decompensated aortic stenosis, atrial fibrillation, myocarditis, and healthy controls were treated with a lysis buffer and subsequently sonicated. Treated and untreated samples were assessed and compared to a conventional EV isolation method. **Results:** Following combined lysis and sonication, cTnT levels were significantly higher compared to native, unprocessed samples across all cohorts. The median increase post-processing ranged from ~10% in decompensated aortic stenosis to ~34% in young healthy controls. In NSTEMI, the EV-bound cTnT accounted for ~15% of plasma cTnT and remained stable over 72 h. The EV cTnT/plasma cTnT ratios were comparable between the combined lysis and sonication approach and the EV isolation method. Processing time prior to cTnT measurement was reduced from ~2.5 h to ~10 min using the lysis and sonication protocol. **Conclusions:** Our method allows for the rapid liberation of a previously inaccessible EV-bound fraction of cTnT without the need for time-consuming and resource-intensive EV isolation workflows and is therefore readily implementable alongside standard hs-cTnT testing. The observed EV-cTnT patterns suggest differential compartmentation of cTnT, potentially reflecting the myocardial pathophysiology underlying troponin elevation.

## 1. Introduction

High-sensitivity cardiac troponin (hs-cTn) assays are widely used in routine clinical diagnostics to detect myocardial injury [1]. However, due to the high analytical sensitivity, even minor or transient troponin elevations related to myocardial pathologies other than overt myocardial necrosis are detected, thereby complicating the clinical interpretation and often necessitating resource-intensive further diagnostics for therapeutic decision-making [2]. As a result, a growing need in clinical routine calls for more specific cardiac biomarkers or the improved characterization of circulating cardiac troponin pools.

Extracellular vesicles (EVs) are nano-sized particles that are secreted into the extracellular space by nearly all cell types [3], transporting a diverse array of molecular cargo, including proteins, lipids, and nucleic acids [4]. As their cargo is enclosed by a lipid bilayer, which ensures stability in circulation, EVs have emerged as promising candidates for liquid biopsy applications across several medical specialties [4,5]. Prior studies have indicated that myocardial ischemia may stimulate EV release [6,7,8,9], and circulating EVs have been associated with altered intravesicular cargo in various cardiovascular diseases [9,10,11,12], generating an increasing scientific and clinical interest in EV-bound cargo as potential cardiac biomarkers in a previously undisclosed blood compartment. However, relatively little attention to date has been drawn to cardiomyocyte-derived EV-bound cardiac troponins. Lennon et al. detected cardiac troponin T within EVs using quantitative single-molecule localization microscopy and found differences in the EV-bound cTnT concentrations and EV size between healthy individuals and patients with heart failure or myocardial infarction [13]. A previous study from our group found that relative EV-bound cTnT signatures could robustly distinguish type I myocardial infarction from cTnT elevation in the context of arrhythmia-induced myocardial strain [14], a clinically highly relevant distinction that cannot reliably be achieved using the current serum or plasma hs-cTnT assays alone. This summative evidence indicates that EV-enclosed cardiac troponins may constitute a previously underappreciated fraction of the total circulating troponin that remains largely inaccessible to routine blood troponin quantification protocols [14,15].

The advantage of hs-cTn assays lies in the rapid diagnosis of acute myocardial infarction [1,16], whereas conventional methods for EV isolation are both time-consuming, requiring several hours, and resource-intensive, limiting their applicability in the acute clinical setting. Against this background, we developed a combined lysis- and sonication-based protocol to disrupt EV membranes and release the EV-bound cTnT in an intact and complete manner, without compromising the speed of the routine hs-cTn measurement and validated our novel method in a real-world cohort of 98 individuals. Our combined lysis and sonication method enables the quantification of a novel biomarker termed ‘total cTnT post EV disruption’, encompassing both free and EV-bound cTnT fractions using a routine, state-of-the-art hs-cTnT immunoassay without prior EV isolation, allowing for the rapid translation to a high-throughput clinical setting with minimal additional analysis time and cost. We hypothesize that measuring the EV-bound cTnT may provide new insights into the mechanisms underlying troponin elevation, potentially enabling the early differentiation of the pathophysiological cause of myocardial injury.

## 2. Materials and Methods

### 2.1. Patient Recruitment

Patients were recruited from the Chest Pain Unit of University Hospital Heidelberg, Germany, following informed consent. Patient recruitment and all subsequent analyses were conducted in accordance with the Declaration of Helsinki in its latest amendment and following approval of the ethics committee at the Medical Faculty of Heidelberg University (IRB approval S-351/2015). A total of 98 participants were included: 32 patients with non-ST-elevation myocardial infarction (NSTEMI), 24 patients with atrial fibrillation (AFib), 11 patients with myocarditis, 5 patients with unstable angina (UA), 5 patients with pulmonary embolism (PE), and 3 patients with decompensated aortic valve stenosis (dAS), as well as 12 young healthy controls and 6 age-matched healthy controls with no prior history of cardiovascular disease. All diagnoses were made or excluded in accordance with current clinical guidelines. Baseline characteristics of the method validation cohort and the full study cohort are summarized in Table 1 and Table 2.

Peripheral venous blood was drawn by routine phlebotomy or via an existing intravenous catheter in lithium heparin and sodium citrate tubes. In patients admitted to the hospital, additional samples were collected every 24 h for up to four time points. Blood samples were immediately stored at 4 °C and processed within 4 h of collection.

### 2.2. Plasma and EV Isolation

Plasma was separated from cellular components of whole blood samples by centrifugation at 750× *g* for 15 min at 4 °C. EVs were isolated from plasma using the Total Exosome Precipitation Kit (Thermo Fisher, Waltham, MA, USA) according to the manufacturer’s instructions.

### 2.3. Combined Lysis and Sonication and cTnT Quantification

Different lysis buffers and buffer concentrations were tested to optimize fast and complete EV lysis and EV-bound cTnT recovery. The lysis buffer and concentration with the highest and fastest yield were selected for combination with low- or high-intensity sonication. Plasma samples were mixed 1:1 (*v*/*v*) with sonication buffer containing 0.1% Triton X-100 (Sigma-Aldrich, St. Louis, MO, USA) and 1% protease/phosphatase inhibitor (Cell Signaling, Danvers, MA, USA) and incubated on ice for 5 min prior to sonication. Sonication was performed in a 4 °C water bath using the Bioruptor Plus (Diagenode, Liège, Belgium) for five cycles of 30 s ON and 30 s OFF at low intensity. Sonicated and non-sonicated plasma samples, EV isolates, EV-depleted plasma, and spiked quality control samples (healthy control plasma supplemented with 100 pg/mL recombinant human cardiac troponin T; HyTest, Turku, Finland) were analyzed using the fifth-generation Elecsys hs-cTnT assay (Roche Diagnostics, Rotkreuz, Switzerland) on the Cobas e402 and e801 analyzers at the central laboratory of University Hospital Heidelberg.

### 2.4. Nanoparticle Tracking Analysis

EV isolates were diluted in PBS to a suitable working solution, resulting in a working concentration of 10–100 particles per frame, and quantified on the NanoSight NS300 (Malvern Panalytical, Malvern, UK), equipped with a 405 nm laser, using NTA 3.2 software under constant acquisition settings (22 °C, camera level 9, detection threshold 2). For each sample, nanoparticle concentration and size were averaged across three 30 s videos.

### 2.5. Immunoblotting

EV isolates and EV-depleted plasma were mixed with Laemmli buffer and 1% (*v*/*v*) beta-mercaptoethanol and denatured at 95 °C for 5 min. Equal sample volumes were loaded onto 4–12% polyacrylamide gels (Bio-Rad, Hercules, CA, USA), followed by gel electrophoresis at constant voltage. Proteins were blotted onto a polyvinylidene difluoride membrane (Bio-Rad) and blocked with 3% bovine serum albumine. Primary antibody (anti-CD9, Cell Signaling, 1:1000) was added and incubated overnight at 4 °C under constant agitation. Secondary antibody (anti-rabbit IgG, horseradish peroxidase-linked, Cell Signaling, 1:1000) was incubated for one hour at room temperature under constant agitation. Membranes were washed before and after adding secondary antibody. For chemiluminescent detection, membranes were incubated with SuperSignal West Femto Maximum Sensitivity solution (Thermo Fisher), protected from light. Imaging was performed on the Fusion FX imager (Vilber Lourmat, Collégien, France), and images were analyzed using NIH ImageJ version 1.53.

### 2.6. Statistical Analysis

Statistical analyses were performed in GraphPad Prism version 9.5.0 and R version 4.6.0. The sample size n represents the number of biological replicates, unless otherwise stated. Normality was assessed using the Shapiro–Wilk test. For normally distributed data, a one-way ANOVA with Bonferroni post hoc correction was used for comparative analysis of three or more groups. Repeated-measures one-way ANOVA with Geisser–Greenhouse correction and Dunnett’s post hoc test was used for comparative analysis of repeated measurements across three or more groups. The paired *t*-test was used for comparisons between paired samples. The Kruskal–Wallis test with Dunn’s post hoc correction was used for comparative analysis of three or more groups for non-normally distributed data. Mann–Whitney U test was used for comparisons between two independent groups. The Wilcoxon matched-pairs signed-rank test was used for paired comparisons. Fold-change ratios (sonicated/native) were tested against a median of 1.0 using the one-sample Wilcoxon signed-rank test. To account for multiple comparisons across cohorts, *p*-values were adjusted using the Benjamini–Hochberg correction and are presented as q-values. Passing–Bablok regression and Bland–Altman analysis were used to compare the novel combined lysis and sonication method with the conventional EV isolation method. Data are presented as mean ± SD unless otherwise stated, and all tests were two-tailed. A *p*-value < 0.05 was considered statistically significant.

## 3. Results

### 3.1. Method Validation

**Figure 1:**
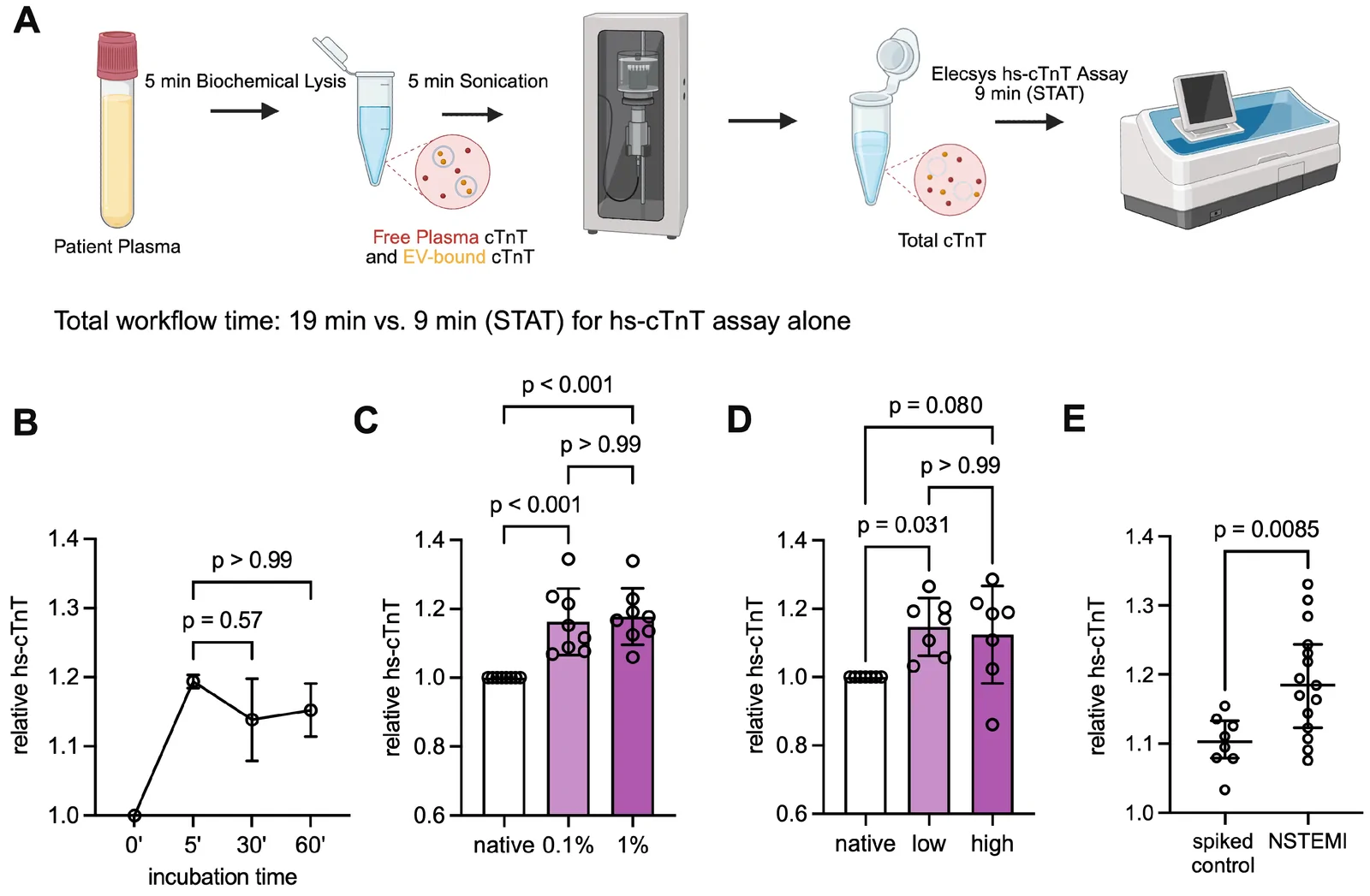
Sonication protocol validation. (**A**) Workflow schematic of biochemical and mechanical lysis of EVs prior to cTnT measurement using a conventional hs-cTnT immunoassay. Created in BioRender under publication license OG29IXGKDC. (**B**) Line plot of relative total hs-cTnT compared to native cTnT at time points 5, 30, and 60 min post combined lysis and sonication. Data shown as mean ± SD, one-way ANOVA with Bonferroni post hoc test, n = 3 biological replicates. (**C**) Relative total hs-cTnT compared to native cTnT after incubation with 0.1% and 1% Triton X-100 and subsequent sonication. Data shown as mean ± SD, one-way ANOVA with Bonferroni post hoc test, n = 8 biological replicates. (**D**) Relative total hs-cTnT compared to native cTnT after lysis and sonication at low and high intensity. Data shown as mean ± SD, one-way ANOVA with Bonferroni post hoc test, n = 7 biological replicates. (**E**) Relative total hs-cTnT in recombinant cTnT-spiked control vs. NSTEMI samples following combined lysis and sonication. Data shown as median [IQR], Mann–Whitney U test, n = 8 biological replicates for spiked controls, and n = 15 biological replicates for NSTEMI.

All relevant parameters of the combined lysis and sonication protocol to release intravesicular cTnT (Figure 1A), including incubation time, lysis buffer concentration, and sonication intensity, were tested for EV-bound cTnT release efficiency. First, the prolonged incubation of samples with a sonication buffer to biochemically lyse EVs had no additive effect with respect to the released cTnT beyond 5 min of incubation (5′ vs. 30′: *p* = 0.57, 5′ vs. 60′: *p* > 0.99, one-way ANOVA with Bonferroni post hoc test, n = 3 biological replicates; Figure 1B), demonstrating that 5 min of incubation prior to sonication is sufficient to release the EV-bound cTnT. Second, increasing the concentration of Triton X-100 from 0.1% to 1% resulted in no additional cTnT increase compared to the native control (*p* > 0.99, one-way ANOVA with Bonferroni post-hoc test, n = 8 biological replicates; Figure 1C). Moreover, no significant difference in the cTnT increase compared to the native control was observed between low and high sonication intensities (*p* > 0.99, one-way ANOVA with Bonferroni post hoc test, n = 7 biological replicates; Figure 1D), suggesting that low-intensity sonication is sufficient to disrupt EV membranes in plasma samples. Lastly, lysis and sonication of plasma from NSTEMI patients led to a significantly higher increase in hs-cTnT levels compared to healthy control plasma spiked with recombinant cTnT (*p* = 0.0085, Mann–Whitney U test, n = 15 NSTEMI patients vs. n = 8 spiked controls; Figure 1E), suggesting that the observed increase may reflect the release of cTnT from the blood EV compartment, rather than protein fragmentation induced by lysis or sonication.

**Figure 2:**
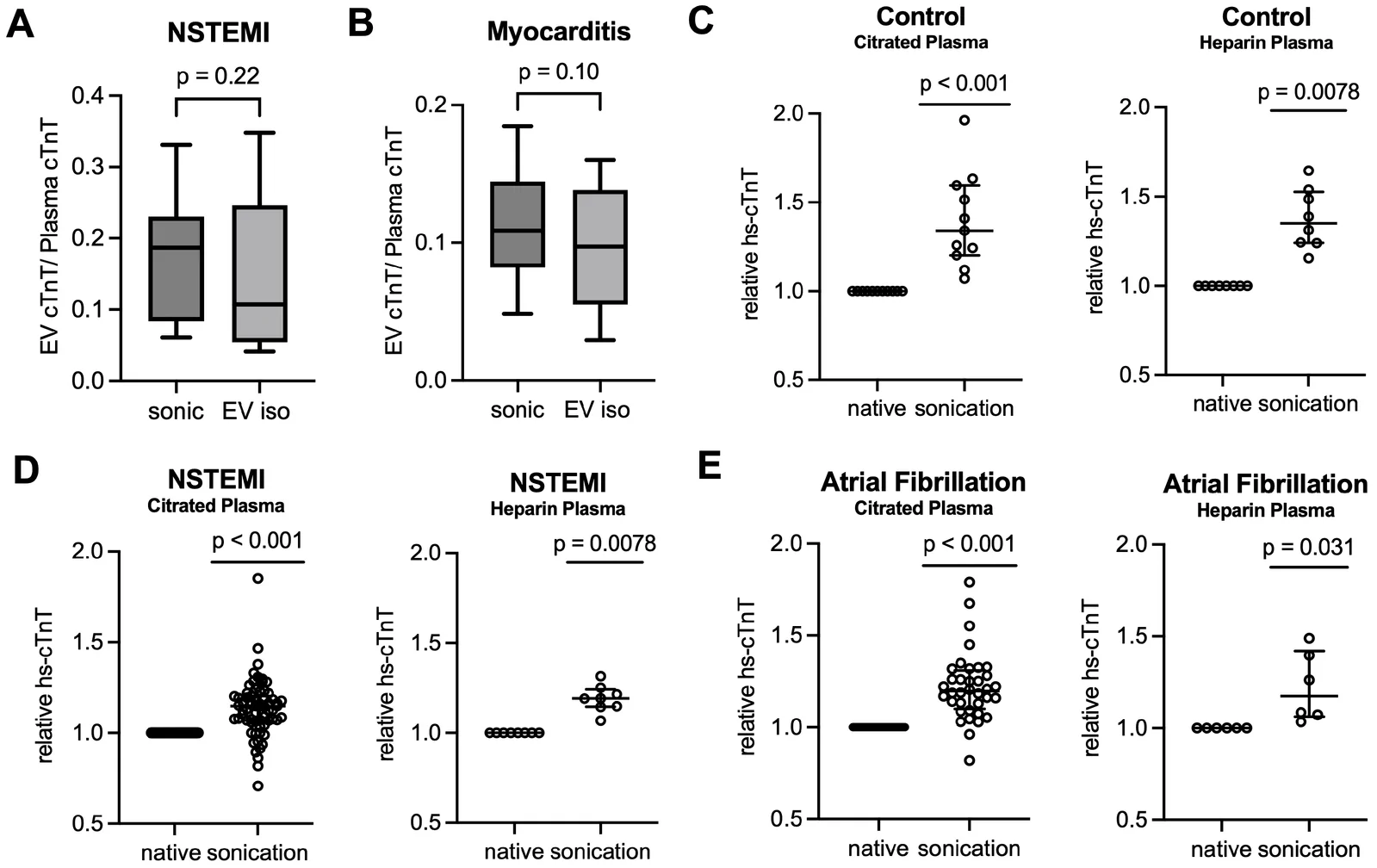
Comparative analysis of combined lysis and sonication with conventional EV isolation in NSTEMI and myocarditis patients, and validation in heparin- and citrate-supplemented blood samples. (**A**) NSTEMI: EV cTnT/plasma cTnT ratio following lysis and sonication (sonic) compared to kit-based EV isolation (EV iso) prior to cTnT measurement. Data displayed as median [IQR], Wilcoxon matched-pairs signed-rank test, n = 13 biological replicates. (**B**) Myocarditis: EV cTnT/plasma cTnT ratio following lysis and sonication compared to kit-based EV isolation prior to cTnT measurement. Data displayed as median [IQR], Wilcoxon matched-pairs signed-rank test, n = 11 biological replicates. (**C**) **Left:** Control, citrated plasma: relative total hs-cTnT post combined lysis and sonication compared to native, unprocessed plasma. Data displayed as median [IQR], one-sample Wilcoxon signed-rank test, n = 11 biological replicates. **Right:** Control, heparin plasma: relative total hs-cTnT post combined lysis and sonication compared to native, unprocessed plasma. Data displayed as median [IQR], one-sample Wilcoxon signed-rank test, n = 8 biological replicates. (**D**) **Left:** NSTEMI, citrated plasma: relative total hs-cTnT post combined lysis and sonication compared to native, unprocessed plasma. Data displayed as median [IQR], one-sample Wilcoxon signed-rank test, n = 67 independent observations from 26 biological replicates, including serial time-point samples. **Right:** NSTEMI, heparin plasma: relative total hs-cTnT post combined lysis and sonication compared to native, unprocessed plasma. Data displayed as median [IQR], one-sample Wilcoxon signed-rank test, n = 8 biological replicates. (**E**) **Left:** Atrial fibrillation, citrated plasma: relative total hs-cTnT post combined lysis and sonication compared to native, unprocessed plasma. Data displayed as median [IQR], one-sample Wilcoxon signed-rank test, n = 36 independent observations from 24 biological replicates, including serial time-point samples. **Right:** Atrial fibrillation, heparin plasma: relative total hs-cTnT post combined lysis and sonication compared to native, unprocessed plasma. Data displayed as median [IQR], one-sample Wilcoxon signed-rank test, n = 6 biological replicates.

To assess the combined lysis and sonication protocol in terms of its ability to release EV-bound cTnT, we performed a comparative analysis using a kit-based EV isolation method, in which EVs were isolated and lysed prior to cTnT measurement. There was no significant difference in hs-cTnT between native and EV-depleted plasma in patients with NSTEMI (*p* > 0.99, Wilcoxon matched-pairs signed-rank test, n = 13 biological replicates; Figure S1A) and myocarditis (*p* = 0.90, Wilcoxon matched-pairs signed-rank test, n = 11 biological replicates; Figure S1E). This suggests that EV-associated cTnT goes largely undetected by conventional immunoassays without prior EV membrane disruption. Accordingly, the ratio of EV-bound cTnT to native plasma cTnT was not significantly different from the ratio of EV-bound cTnT to EV-depleted plasma cTnT in either cohort (NSTEMI: *p* = 0.17, Wilcoxon matched-pairs signed-rank test, n = 13 biological replicates; Figure S1D. Myocarditis: *p* = 0.15, Wilcoxon matched-pairs signed-rank test, n = 11 biological replicates; Figure S1H). In the NSTEMI samples, the concentrations of EV-bound cTnT after lysis and sonication were significantly higher than those measured with the kit-based method (*p* = 0.040, Wilcoxon matched-pairs signed-rank test, n = 13 biological replicates; Figure S1C). In myocarditis samples, a similar trend was observed (*p* = 0.067, Wilcoxon matched-pairs signed-rank test, n = 11 biological replicates; Figure S1G). Notably, the total measurable cTnT after combined lysis and sonication was significantly higher than the sum of the EV-bound cTnT and native plasma cTnT detected after kit-based isolation in both cohorts (NSTEMI: *p* = 0.040, Wilcoxon matched-pairs signed-rank test, n = 13 biological replicates; Figure S1B. Myocarditis: *p* = 0.032, Wilcoxon matched-pairs signed-rank test, n = 11 biological replicates; Figure S1F). These findings may reflect a certain degree of loss of cTnT-containing EVs during the precipitation-based EV isolation, and further point towards a more complete EV lysis in suspension and EV-bound cTnT release with the combined lysis and sonication protocol. No significant difference in the EV cTnT/plasma cTnT ratio was detected between the two methods (NSTEMI: *p* = 0.22, Wilcoxon matched-pairs signed-rank test, n = 13 biological replicates; Figure 2A. Myocarditis: *p* = 0.10, Wilcoxon matched-pairs signed-rank test, n = 11 biological replicates; Figure 2B). Passing–Bablok regression demonstrated good agreement between the combined lysis and sonication approach and the conventional EV isolation method for both absolute total cTnT post EV disruption and the EV cTnT/plasma cTnT ratio (pooled cohorts: n = 24 biological replicates; Figure S2). For absolute total cTnT post disruption, the regression fit showed an intercept of −2.73 (95% CI: −6.74 to −0.05) and a slope of 1.05 (95% CI: 1.02 to 1.11; Figure S2A), with a mean bias of 6.0 ng/L (95% limits of agreement: −11 to 23 ng/L; Figure S2B). For EV cTnT/plasma cTnT ratios, the intercept was 0.031 (95% CI: −0.010 to 0.058) and the slope 0.881 (95% CI: 0.608 to 1.536; Figure S2C), with a mean bias of 0.023 (95% limits of agreement: −0.096 to 0.14; Figure S2D). Overall, these results indicate that combined lysis and sonication achieved a non-inferior efficacy with respect to EV-bound cTnT release without the need for prior EV isolation from plasma.

Using nanoparticle tracking analysis, a significant decrease in relative EV concentration was observed following combined lysis and sonication (before: 3.77 × 10^9^ ± 2.32 × 10^9^ particles/mL, after: 1.57 × 10^9^ ± 7.23 × 10^8^ particles/mL, *p* = 0.016, Mann–Whitney U test, n = 5 biological replicates; Figure S3A), suggesting successful disruption of EVs, while no significant change in the mean EV size was found (before: 101.2 ± 31.2 nm, after: 124.7 ± 32.6 nm, *p* = 0.31, Mann–Whitney U test, n = 5 biological replicates; Figure S3B). In accordance with the nanoparticle tracking data, immunoblotting for the canonical EV marker CD9 revealed a significantly lower expression in processed samples compared to native samples, further indicating effective disruption of EVs after lysis and sonication (*p* = 0.037, paired *t*-test, n = 3 biological replicates; Figure S4).

We also tested whether our protocol using citrate-supplemented blood as the starting material could be applied to heparin plasma samples. Combined lysis and sonication led to a significant hs-cTnT increase in samples from healthy controls (median fold change = 1.34, *p* = 0.0078, q = 0.011, one-sample Wilcoxon signed-rank test, n = 8 biological replicates; Figure 2C, right panel), NSTEMI patients (median fold change = 1.19, *p* = 0.0078, q = 0.011, one-sample Wilcoxon signed-rank test, n = 8 biological replicates; Figure 2D, right panel), and atrial fibrillation patients (median fold change = 1.17, *p* = 0.031, q = 0.031, one-sample Wilcoxon signed-rank test, n = 6 biological replicates; Figure 2E, right panel). A direct comparison of post-lysis and -sonication fold changes showed no significant differences between the citrate and heparin plasma samples in either cohort (healthy controls: *p* > 0.99; NSTEMI: *p* = 0.35; atrial fibrillation: *p* > 0.99; Mann–Whitney U test), suggesting that our combined lysis and sonication protocol may also be applicable to heparin plasma samples.

### 3.2. Combined Lysis and Sonication Reveals Potential EV-cTnT Patterns Depending on Underlying Cardiac Pathophysiology

Lysis and sonication of citrate-supplemented plasma samples resulted in increased hs-cTnT levels compared with native, unprocessed plasma across all cohorts. The study cohort consisted of patients with NSTEMI, unstable angina, pulmonary embolism, decompensated aortic stenosis, atrial fibrillation, myocarditis, and healthy controls. Patients with cardiovascular disease were, on average, older, had a higher BMI, and lower renal function compared with young healthy controls (Table 2). To account for potential confounding, an additional age-matched control group was included. As expected, cardiac troponin levels were significantly higher in patients with cardiovascular disease, consistent with ongoing cardiac pathophysiology upon presentation. Similar tendencies were observed in the validation cohort (Table 1).

**Figure 3:**
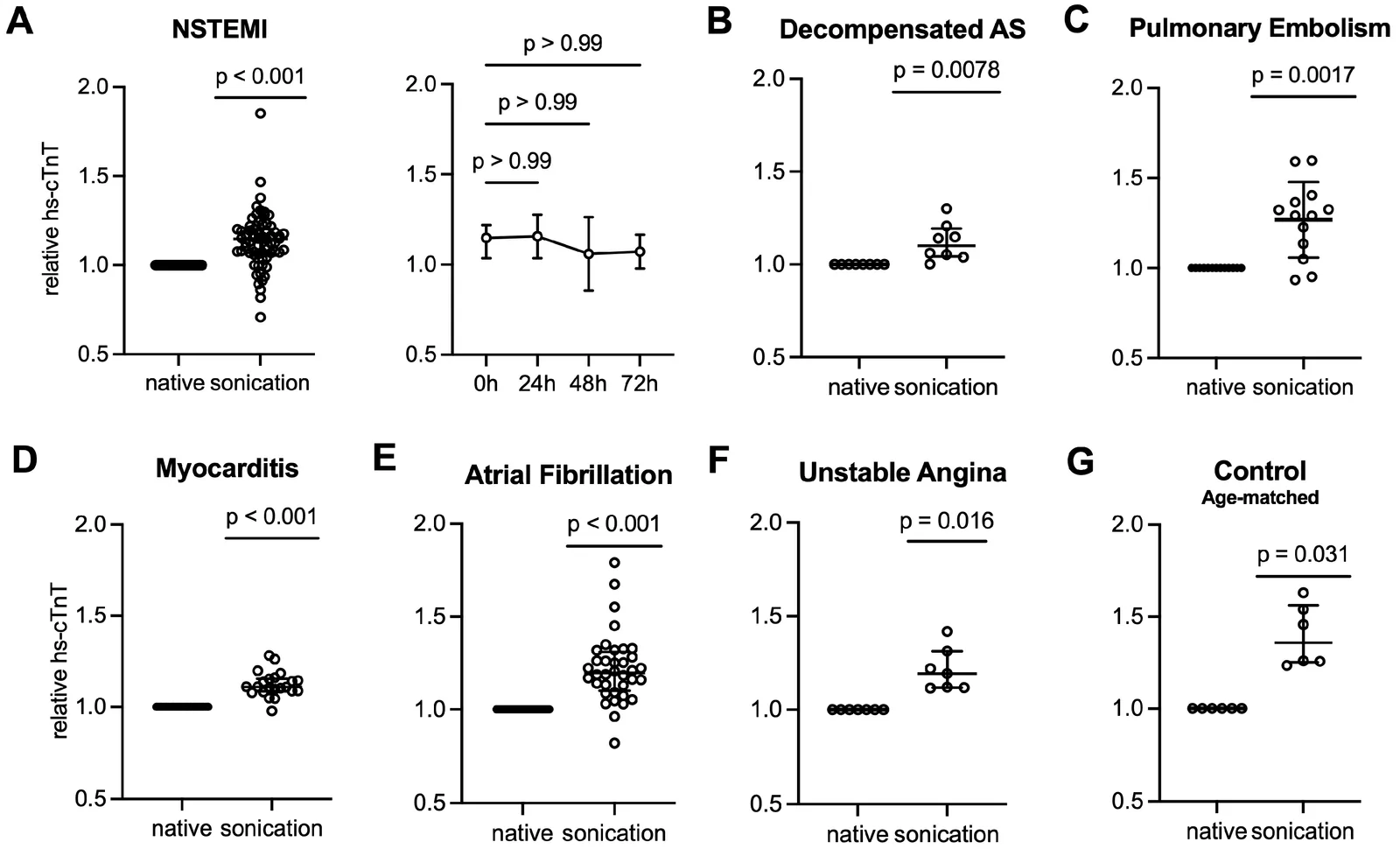
(**A**) NSTEMI: **Left:** Relative total hs-cTnT post combined lysis and sonication compared to native, unprocessed plasma. Data displayed as median [IQR], one-sample Wilcoxon signed-rank test, n = 67 independent observations from 26 biological replicates, including serial time-point samples. **Right:** Line plot of relative total hs-cTnT of serial NSTEMI samples at 0, 24, 48, and 72 h post presentation to the emergency department after combined lysis and sonication. Data displayed as median [IQR], Kruskal–Wallis with Dunn’s post hoc test, n = 13, 15, 11, and 6 biological replicates, respectively. (**B**) Decompensated aortic stenosis (dAS): Relative total hs-cTnT post combined lysis and sonication compared to native, unprocessed plasma. Data displayed as median [IQR], one-sample Wilcoxon signed-rank test, n = 8 independent observations from 3 biological replicates, including serial time-point samples. (**C**) Pulmonary embolism: Relative total hs-cTnT post combined lysis and sonication compared to native, unprocessed plasma. Data displayed as median [IQR], one-sample Wilcoxon signed-rank test, n = 13 independent observations from 5 biological replicates, including serial time-point samples. (**D**) Myocarditis: Relative total hs-cTnT post combined lysis and sonication compared to native, unprocessed plasma. Data displayed as median [IQR], one-sample Wilcoxon signed-rank test, n = 22 independent observations from 11 biological replicates, including serial time-point samples. (**E**) Atrial fibrillation: Relative total hs-cTnT post combined lysis and sonication compared to native, unprocessed plasma. Data displayed as median [IQR], one-sample Wilcoxon signed-rank test, n = 36 independent observations from 24 biological replicates, including serial time-point samples. (**F**) Unstable angina: Relative total hs-cTnT post combined lysis and sonication compared to native, unprocessed plasma. Data displayed as median [IQR], one-sample Wilcoxon signed-rank test, n = 7 independent observations from 5 biological replicates, including serial time-point samples. (**G**) Age-matched control: Relative total hs-cTnT post combined lysis and sonication compared to native, unprocessed plasma. Data displayed as median [IQR], one-sample Wilcoxon signed-rank test, n = 6 biological replicates.

Following combined lysis and sonication, a median increase from the native, unprocessed baseline of approximately 15% was observed in NSTEMI samples (*p* < 0.001 vs. baseline, q = 0.0002, one-sample Wilcoxon signed-rank test, n = 67 independent observations; Figure 3A, left panel), suggesting that the EV-bound cTnT accounts for approximately 15% of the plasma cTnT measured in clinical routine, concurrent with previous data [14]. Serial sampling showed no significant change in relative EV-bound cTnT levels over the first 72 h following presentation to the emergency department (0 h vs. 24 h: *p* > 0.99, 0 h vs. 48 h: *p* > 0.99, 0 h vs. 72 h: *p* > 0.99; Kruskal–Wallis with Dunn’s post hoc test, n = 13 vs. 15 vs. 11 vs. 6 biological replicates per group, respectively; Figure 3A, right panel). In plasma samples from patients with decompensated aortic stenosis, a median increase of approximately 10% compared to the unprocessed baseline was found (*p* = 0.0078, q = 0.010, one-sample Wilcoxon signed-rank test, n = 8 independent observations; Figure 3B). In pulmonary embolism, the largest increase of approximately 29% following combined lysis and sonication was observed among all disease groups (*p* = 0.0017, q = 0.0027, one-sample Wilcoxon signed-rank test, n = 13 independent observations; Figure 3C), followed by atrial fibrillation (~20%, *p* < 0.001, q = 0.0002, one-sample Wilcoxon signed-rank test, n = 36 independent observations; Figure 3E), unstable angina (~19%, *p* = 0.016, q = 0.018, one-sample Wilcoxon signed-rank test, n = 7 independent observations; Figure 3F), and myocarditis (~11%, *p* < 0.001, q = 0.0002, one-sample Wilcoxon signed-rank test, n = 22 independent observations; Figure 3D). In accordance with our previous data [14], young healthy controls showed the highest increase in hs-cTnT following combined lysis and sonication at approximately 34% of the native plasma (*p* < 0.001, q = 0.0002, one-sample Wilcoxon signed-rank test, n = 11 biological replicates; Figure 2C, left panel). In the age-matched healthy controls, an increase in hs-cTnT of approximately 36% was observed (*p* = 0.031, q = 0.031, one-sample Wilcoxon signed-rank test, n = 6 biological replicates; Figure 3G). Of note, the increase in hs-cTnT following lysis and sonication remained statistically significant across all cohorts after correction for multiple comparisons. These findings suggest that, under physiological conditions, the relative EV-bound fraction of circulating cTnT accounts for approximately one-third of free plasma cTnT, potentially reflecting baseline EV secretion by the myocardium under cardiac homeostasis.

## 4. Discussion

Recently, we reported on the presence of EV-bound cardiac troponin in different disease states, including NSTEMI, but also in conditions other than acute coronary syndromes where other pathomechanisms are suspected, including transient pathologies that do not lead to irreversible cardiomyocyte death [14]. We also found different compartmentation patterns and EV/blood ratios of cardiac troponins across cardiovascular diseases [14].

Unfortunately, the quantification of EV-bound cardiac troponin requires time- and labor-intensive analytical processes. Therefore, we herein introduce a new method that combines lysis with sonication. In particular, we tried to find a protocol that allowed the shortest pre-analytical time without compromising the integrity of cardiac troponin. For this purpose, different buffers and buffer concentrations were tested, with or without low or high sonication.

Our goal was to develop a novel lysis- and sonication-based protocol that enables the release and quantification of EV-bound cTnT in human plasma samples using a conventional hs-cTnT immunoassay without prior EV isolation. Importantly, this methodological approach decouples EV-bound cTnT quantification from conventional EV isolation workflows, enabling direct implementation within existing clinical workflows with minimal additional time and cost. The individual steps of the combined lysis and sonication protocol were independently tested and optimized for efficiency. Moreover, our validation studies provide evidence for the near-complete EV lysis with cTnT liberation, as well as largely exclude troponin fragmentation and the subsequent multilocular detection as the cause of the observed increase in hs-cTnT with relative diagnostic certainty.

In the synopsis, we report seven important findings:

First, combined lysis and sonication enables the complete release of EV-bound cargo, such as cTnT, into free plasma, thereby rendering a hitherto inaccessible pool measurable without the need for time- and labor-consuming EV isolation.

Second, the yield of EV-cTnT is at least as high with the lysis and sonication method as with the EV isolation method. In this context, low sonication proved equally effective as high sonication, reducing potential mechanical damage to EVs and their cargo.

Third, our data suggest that combined lysis and sonication does not affect the recovery of cTnT; i.e., it does not interfere with epitope detection through conformational changes or degradation by either the lysis buffer or mechanical damage from sonication.

Fourth, after a single lysis and sonication step consuming around 10 min, the recovery is maximal, reaching at least the same concentrations of EV-cTnT as with the EV isolation method. As such, no waiting times for incubation or other lysis-related processes are required. Hence, combined lysis and sonication alongside the standard troponin measurement on an automated platform could become an attractive approach for measuring EV-bound troponin in clinical routine.

Fifth, the lysis and sonication protocol requires a processing time of approximately 10 min vs. approximately 2.5 h for the EV isolation method used in this study, and thus appears promising for use in clinical practice, even for suspected NSTE-ACS, where time is crucial for early diagnosis and effective treatment. We speculate that integration into existing laboratory workflows could be feasible through either the sequential measurement of a single blood sample, i.e., one conventional run followed by a second measurement after lysis and sonication, or the simultaneous measurement on two analyzers, at an additional time expense of 10 min on top of the standard STAT turnaround time of 9 min [17], depending on the laboratory infrastructure and time constraints.

Sixth, in long-term storage and freeze–thaw stability experiments, we show that EV-bound cTnT remains stable in blood specimens frozen at −80 °C for up to 30 days (*p* = 0.98, repeated-measures one-way ANOVA with Geisser–Greenhouse correction and Dunnett’s post hoc test, n = 5 biological replicates; Figure S5A) and for up to five freeze–thaw cycles (*p* = 0.095, repeated-measures one-way ANOVA with Geisser–Greenhouse correction and Dunnett’s post hoc test, n = 4 biological replicates; Figure S5B). Ongoing experiments are evaluating the stability over longer time periods in frozen plasma samples and frozen EV isolates. As such, the new method for the measurement of EV-derived cargo—including but not limited to cardiac troponin—is anticipated to facilitate routine laboratory testing without the need for the time-consuming isolation of EVs from fresh blood and might open a new avenue for the analysis of stored frozen blood from existing biobanks.

Seventh, our previous findings on the differential compartmentation of EV-bound cardiac troponin across pathophysiological states, and the ability to detect EV-associated troponin that has not undergone conformational changes or degradation in peripheral blood, generate the hypothesis that this approach may provide additional information for interpreting hs-cTn results in patients with acute or chronic myocardial injury, warranting confirmation in future diagnostic accuracy studies. Moreover, this approach may prove valuable in conditions where conventional blood-based methods are ineffective or where the target molecules are prone to degradation or conformational changes in blood, such as cardiac amyloidosis or chemotherapy-related cardiotoxicity.

In the present study, the majority of circulating cTnT in NSTEMI patients was present in the free plasma fraction, with approximately 15% associated with the EV compartment, consistent with our prior study on relative EV-cTnT through separate compartmental analysis of plasma-derived EV and free plasma fractions [14]. This compartmentation of cTnT remained stable in the first 72 h following initial presentation. Whether this observation indicates continuous EV secretion from stressed myocardium or the prolonged stability of EV-bound cTnT in circulation remains unclear. Future studies are needed to investigate the kinetics of EV-cTnT secretion and degradation to help distinguish between these possibilities. Findings in this method study consistently confirm our previously reported observations using the established EV isolation method, i.e., that healthy controls show the highest EV-bound cTnT fraction [14], suggesting that, under physiological conditions, in the absence of cardiomyocyte necrosis, a larger proportion of circulating troponin may be EV-associated than in diseased states, and free-plasma cTnT may originate from the EV fraction due to natural EV degradation. Likewise, in conditions of transient myocardial strain, such as atrial fibrillation or pulmonary embolism, an intermediate relative EV-bound fraction of cTnT between that observed in acute myocardial infarction and healthy controls was found. In myocarditis, despite a predominantly inflammatory etiology, EV fractions were comparable to those observed in acute ischemia, suggesting an advanced disease stage and substantial cardiomyocyte injury at the time of blood sampling. In summary, measuring the EV-bound cTnT fraction may help distinguish strain from necrosis-driven troponin elevations, a distinction that the current hs-cTnT assays are unable to reliably achieve, confirming our prior work [14] and complementing the findings of Lennon et al., who reported altered EV biology and cTnT compartmentation in heart failure and acute myocardial infarction [13]. Thus, measuring EV-cTnT may enable earlier differentiation of the pathophysiological mechanisms of troponin elevations, complementing existing diagnostic workup strategies.

Beyond its potential diagnostic implications, this study could also serve as preliminary evidence for simplifying plasma-based EV research. Over the last decade, EVs have emerged as a hallmark of biomarker research across various disease entities. A method that enables the quantification of EV cargo without rigorous EV isolation would facilitate protocols for large-scale applications and increase the accessibility of these translational findings in everyday clinical practice. A significant decrease in EV-cTnT recovery was observed at 90 days in long-term storage stability experiments (*p* = 0.007, repeated-measures one-way ANOVA with Geisser–Greenhouse correction and Dunnett’s post hoc test, n = 5 biological replicates; Figure S5A). Combined with the apparent stability of EV-cTnT in freeze–thaw experiments, these findings suggest the applicability of our method to archived biobank samples for this time period, allowing for retrospective analyses. However, given the exploratory nature of these experiments, further studies are needed to confirm these observations.

Several limitations should be acknowledged. First, healthy controls in the primary study cohort were not age-matched to the patient cohorts. Since age is a recognized biological variable influencing EV biology, the relatively higher EV-bound cTnT fraction observed in the healthy controls could reflect age-related differences rather than a state of myocardial homeostasis [18]. However, in a small cohort of age-matched controls, similar EV-bound cTnT fractions were found (~36% vs. ~34% in younger healthy controls, *p* = 0.81, Mann–Whitney U test), suggesting that this observation might not be exclusively explained by age. Nevertheless, given the small sample size, this finding should be interpreted with caution and warrants further investigation in larger cohorts. Furthermore, renal function may also influence circulatory EV kinetics and may thus account for the differences in the relative EV concentration and EV-bound cTnT. Whilst the new control group is age-, weight-, and sex-matched, further differences in renal function could also contribute to the variability in circulating EV and cTnT dynamics and were not systematically adjusted for in the present analysis, potentially confounding the comparisons across cohorts. Moreover, the statistical power was limited by small sample sizes in several subgroups, as the primary aim of this study was to establish a proof-of-concept for the lysis and sonication method across different disease groups, rather than to provide adequately powered comparisons between cohorts. Accordingly, *p*-values from subgroups with n < 10 should be interpreted with caution and regarded as exploratory and of hypothesis-generating nature. Larger, adequately powered studies are needed to confirm these findings and to establish disease-specific reference ranges for total and relative EV-bound cTnT. In addition, several cohorts included serial samples from the same patients, which may introduce within-subject correlation and violate the assumption of full independence in some analyses. To address this circumstance, we performed a sensitivity analysis for the NSTEMI cohort, including only one sample per patient, which yielded results consistent with our primary analysis (median increase: ~14.9%, n = 26 vs. ~14.8%, n = 67; *p* = 0.54, Mann–Whitney U test). However, the potential impact of repeated samples from the same patients should be kept in mind when interpreting our findings. Moreover, this study did not directly assess whether non-EV particles or assay interference contribute to the observed cTnT increase. Additionally, while the increase in blood concentration by EV-cTnT is consistent, we cannot exclude the analytical imprecision at the lower end of the concentration range, which is why our method may be more suitable for evaluating trends or ratios than for a precise quantification at clinical decision thresholds. Forthcoming studies are necessary to investigate whether differences in the EV-bound cTnT pool allow for the differentiation between diseases. Further, mechanistic studies are needed to unveil the mechanism of intracellular structural troponin disintegration, the enrichment in EV, and the subsequent release into circulation by cardiomyocytes, as well as the biology of EV degradation and cTnT release from EV, thus potentially contributing to systemic circulating plasma troponin levels. Chronic troponin elevations observed in conditions such as heart failure or valvular disease may, in part, reflect sustained EV secretion from persistently stressed cardiomyocytes rather than ongoing ischemic injury, a hypothesis that warrants further mechanistic investigation. In addition, this method may open opportunities for the investigation of other EV-bound compounds from fresh or frozen plasma samples that are susceptible to degradation or conformational changes in free plasma, such as proteins involved in cardiac amyloidosis. Finally, this study focused exclusively on cTnT. However, our previous study has shown similar compartmentation patterns of cardiac troponin I in myocardial infarction and atrial fibrillation [14].

In conclusion, our combined lysis and sonication protocol enables the release and quantification of EV-bound cTnT from plasma samples in a time-efficient and cost-effective manner, uncovering potential EV-cTnT patterns in blood. Quantitative analysis of this previously inaccessible fraction alongside conventional hs-cTnT testing may provide additional pathophysiological insight into troponin elevation, ultimately generating the hypothesis that troponin compartmentation may be disease-specific, warranting further investigation.

## Figures and Tables

**Table 1:** Patient demographics of the method validation cohort.

	Control (n = 8)	NSTEMI (n = 12)	*p*-Value
**Age (yrs)**	24.9 (±9.2)	72.0 (±10.2)	<0.0001
**Male gender**	50%	92%	0.11
**BMI** **(kg/m^2^)**	23.7 (±5.0)	29.3 (±6.3)	0.13
**Systolic BP** **(mmHg)**	126.6 (±16.0)	139.4 (±25.7)	0.30
**Heart rate** **(min^−1^)**	80.4 (±7.3)	82.9 (±18.9)	0.81
**Days since onset of symptoms**	N/A	1.3 (±1.6)	N/A
**Serum hs-cTnT** **(pg/mL)**	8.1 (±4.4)	349.8 (±550.4)	<0.0001
**eGFR (mL/min)**	115.0 (±22.0)	79.4 (±19.7)	0.09

**Table 2:** Patient demographics of the study cohort.

	Control (n = 12)	Control Age-Matched (n = 6)	NSTEMI (n = 32)	AFib (n = 24)	Myocarditis (n = 11)	UA (n = 5)	PE (n = 5)	dAS (n = 3)	*p*-Value Control	*p*-Value Control Age-Matched
**Age (yrs)**	28.4 (±10.1)	65.7 (±19.0)	69.1 (±13.2)	70.2 (±10.3)	46.6 (±27.1)	71.0 (±12.0)	56.6 (±17.1)	80.3 (±5.1)	<0.0001	0.11
**Male gender**	69%	50%	78%	79%	82%	100%	60%	100%	0.69	0.42
**BMI** **(kg/m^2^)**	24.0 (±2.5)	23.23 (±3.4)	27.8 (±5.0)	29.2 (±4.8)	25.7 (±3.3)	27.6 (±5.1)	29.5 (±2.8)	27.3 (±4.3)	0.03	0.09
**Systolic BP** **(mmHg)**	122.4 (±13.6)	120.0 (±15.5)	140.1 (±24.1)	128.9 (±20.7)	131.3 (±20.6)	142 (±20.7)	122.8 (±19.3)	142.7 (±16.2)	0.12	0.18
**Heart rate** **(min^−1^)**	71.8 (±9.5)	88.3 (±15.0)	81.4 (±18.7)	96.0 (±28.5)	79.9 (±12.5)	80.8 (±4.9)	81.8 (±24.2)	75.0 (±8.7)	0.11	0.37
**Days since onset of symptoms**	N/A	N/A	1.9 (±5.2)	3.7 (±6.1)	8.5 (±13.5)	2.9 (±6.2)	11.7 (±11.6)	24.0 (±20.3)	N/A	N/A
**Serum hs-cTnT** **(pg/mL)**	7.57 (±4.4)	8.5 (±4.3)	574.7 (±1686.8)	28.4 (±21.4)	451.5 (±579.6)	13.8 (±8.4)	36.0 (±36.7)	33.2 (±12.4)	<0.0001	<0.0001
**eGFR (mL/min)**	116.6 (±20.0)	83.7 (±14.9)	80.7 (±25.0)	61.7 (±14.7)	87.5 (±34.0)	75.4 (±15.2)	86.1 (±28.8)	62.8 (±7.6)	<0.0001	0.02

## Data Availability

The data presented in this study are available upon reasonable request from the corresponding author due to ethical restrictions.

## Supplementary Materials

The following supporting information can be downloaded at: https://www.mdpi.com/article/10.3390/biomedicines14081653/s1, Figure S1: Comparison of combined lysis and sonication to kit-based EV isolation in NSTEMI (A–D, n = 13 biological replicates) and myocarditis plasma samples (E–H, n = 11 biological replicates). (A,E) Hs-cTnT concentrations in native plasma compared to EV-depleted plasma. (B,F) Total hs-cTnT concentrations following combined lysis and sonication compared to the sum of EV-bound and native plasma cTnT following prior EV isolation. (C,G) EV-bound hs-cTnT concentrations following lysis and sonication compared to EV isolation method. (D,H) EV cTnT/plasma cTnT ratio relative to native or EV-depleted plasma cTnT. All data displayed as median [IQR], Wilcoxon matched-pairs signed-rank test. Figure S2: Method comparison of combined lysis and sonication vs. EV isolation. (A) Passing-Bablok regression comparing total troponin (ng/L) using combined lysis and sonication vs. conventional EV isolation (n = 24 biological replicates; NSTEMI n = 13 biological replicates, myocarditis n = 11 biological replicates). The solid line represents the regression fit (intercept: −2.73, 95% CI: −6.74 to −0.05; slope: 1.05, 95% CI: 1.02 to 1.11); the dashed line represents the identity line. (B) Bland-Altman plot comparing total troponin (ng/L) using combined lysis and sonication vs. conventional EV isolation (n = 24 biological replicates; NSTEMI n = 13 biological replicates, myocarditis n = 11 biological replicates). Positive values indicate higher values with combined lysis and sonication compared to EV isolation. The solid line indicates mean bias (6.0 ng/L); dotted lines indicate 95% limits of agreement (−11 to 23 ng/L). (C) Passing-Bablok regression comparing EV cTnT/plasma cTnT ratios using combined lysis and sonication vs. conventional EV isolation (n = 24 biological replicates; NSTEMI n = 13 biological replicates, myocarditis n = 11 biological replicates). The solid line represents the regression fit (intercept: 0.031, 95% CI: −0.010 to 0.058; slope: 0.881, 95% CI: 0.608 to 1.536); the dashed line represents the identity line. (D) Bland-Altman plot comparing EV cTnT/plasma cTnT ratios using combined lysis and sonication vs. conventional EV isolation (n = 24 biological replicates; NSTEMI n = 13 biological replicates, myocarditis n = 11 biological replicates). Positive values indicate higher ratios with combined lysis and sonication compared to EV isolation. The solid line indicates mean bias (0.023); dotted lines indicate 95% limits of agreement (−0.096 to 0.14). Figure S3: Nanoparticle tracking analysis before and after combined lysis and sonication. EVs were isolated from plasma of healthy controls before and after combined lysis and sonication using an EV isolation kit. (A) Relative particle concentration in native vs. processed (sonication) samples. Data are presented as mean ± SD, Mann-Whitney U test, n = 5 biological replicates with three technical replicates each. (B) EV size (nm) of corresponding native and processed (sonication) samples. Data are presented as mean ± SD, Mann-Whitney U test, n = 5 biological replicates with three technical replicates each. Figure S4: Immunoblotting for EV marker CD9 in EV isolates and EV-depleted plasma of healthy controls. (A) Immunoblotting for CD9 in native and processed (sonic) EV isolates (EV) from three biological replicates (1–3), and EV-depleted plasma (ΔEV plasma, replicates 1–3). (B) Quantification of relative CD9 band intensity in native and processed (sonication) EV samples, normalized to native control. Data presented as mean ± SD, paired t-test, n = 3 biological replicates. (C) Full immunoblot of (A) showing specific enrichment of CD9 in native and processed EV isolates, and absence of enrichment in EV-depleted plasma. L = ladder. Figure S5: Storage and freeze-thaw stability of EV-bound cTnT. (A) Citrated plasma of healthy controls was stored at −80 °C. EV were isolated by ultracentrifugation, and EV-bound hs-cTnT was measured immediately after collection and at 7, 30, and 90 days after initial freezing, normalized to the mean at baseline. Data are presented as mean ± SD, Shapiro-Wilk normality test followed by repeated-measures one-way ANOVA with Geisser-Greenhouse correction and Dunnett’s post-hoc test, n = 5 biological replicates. (B) Freeze-thaw stability of EV-bound hs-cTnT after 0, 1, 2, 3, and 5 cycles in plasma from healthy controls. For each cycle, separate aliquots were frozen at −80 °C for at least 2 h and thawed on ice for 1 h, followed by combined lysis and sonication, and normalized to the mean at baseline. Data are presented as mean ± SD, Shapiro-Wilk normality test followed by repeated-measures one-way ANOVA with Geisser-Greenhouse correction and Dunnett’s post-hoc test, n = 4 biological replicates.

## Abbreviations

The following abbreviations are used in this manuscript:

hs-cTn	high-sensitivity cardiac troponin
cTnT	cardiac troponin T
EV	extracellular vesicle
NSTEMI	non-ST-elevation myocardial infarction
UA	unstable angina
dAS	decompensated aortic valve stenosis
PE	pulmonary embolism
AFib	atrial fibrillation
