# Supplementary material for: A Lysis- and Sonication-Based Method for the Quantification of Extracellular Vesicle-Bound Cardiac Troponin T Using a High-Sensitivity Immunoassay
Source: Biomedicines. 2026 Jul 23;14(8):1653. doi: 10.3390/biomedicines14081653 (PMC13509610; doi:10.3390/biomedicines14081653)
Supplement: Supplementary file 1 [file biomedicines-14-01653-s001.zip › biomedicines-4350764-supplementary.pdf]

Supplementary Material

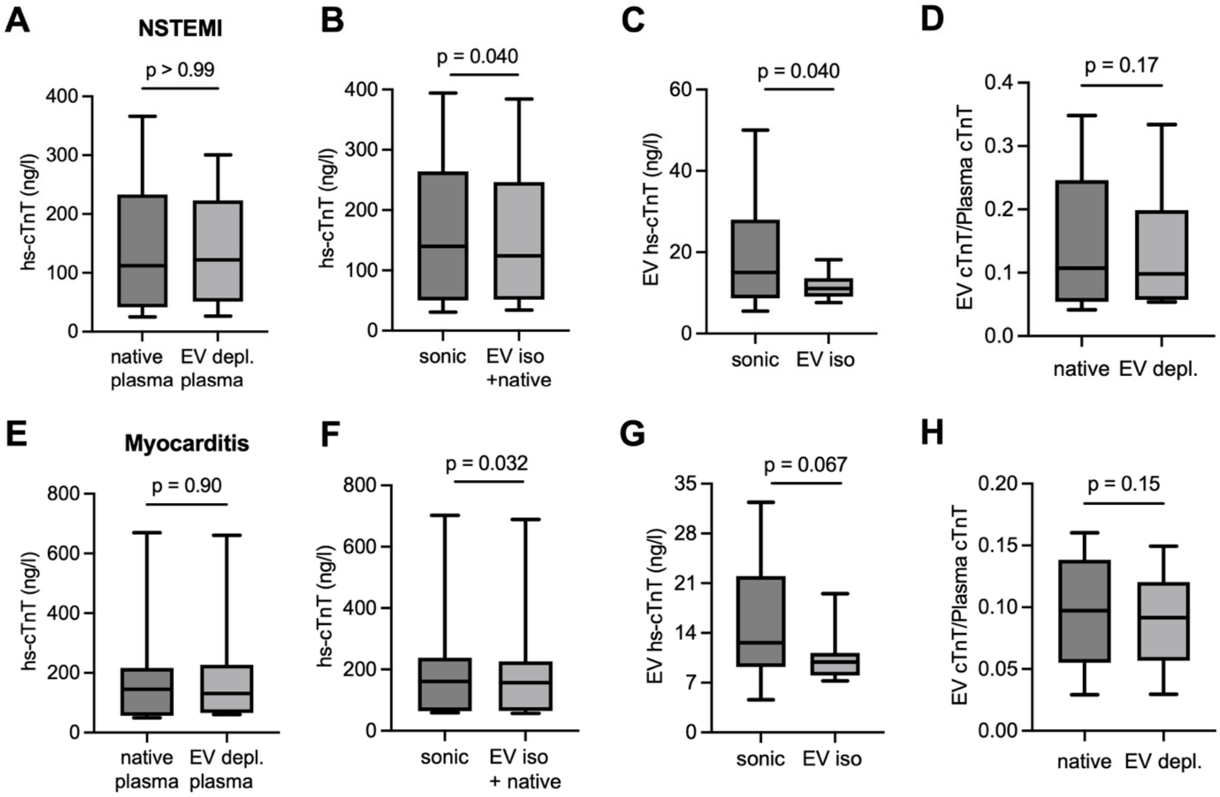

Supplemental Figure S1. Comparison of combined lysis and sonication to kit-based EV isolation in NSTEMI (A-D, n=13 biological replicates) and myocarditis plasma samples (E-H, n=11 biological replicates). (A,E) Hs-cTnT concentrations in native plasma compared to EV-depleted plasma. **(B,F)** Total hs-cTnT concentrations following combined lysis and sonication compared to the sum of EV-bound and native plasma cTnT following prior EV isolation. **(C,G)** EV-bound hs-cTnT concentrations following lysis and sonication compared to EV isolation method. **(D,H)** EV cTnT/plasma cTnT ratio relative to native or EV-depleted plasma cTnT. All data displayed as median [IQR], Wilcoxon matched-pairs signed-rank test.

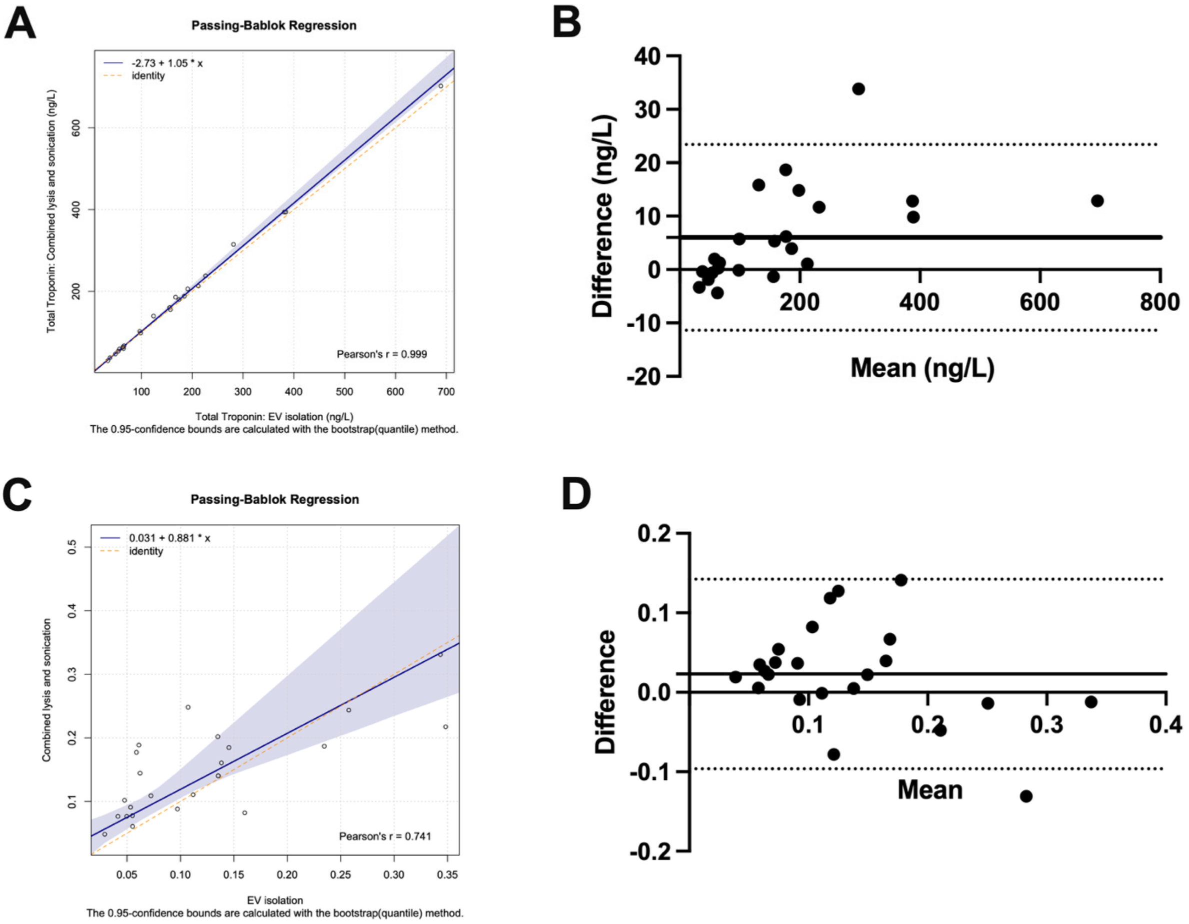

Supplemental Figure S2. Method comparison of combined lysis and sonication vs. EV isolation. (A) Passing-Bablok regression comparing total troponin (ng/L) using combined lysis and sonication vs. conventional EV isolation (n=24 biological replicates; NSTEMI n=13 biological replicates, myocarditis n=11 biological replicates). The solid line represents the regression fit (intercept: −2.73, 95% CI: −6.74 to −0.05; slope: 1.05, 95% CI: 1.02 to 1.11); the dashed line represents the identity line. **(B)** Bland-Altman plot comparing total troponin (ng/L) using combined lysis and sonication vs. conventional EV isolation (n=24 biological replicates; NSTEMI n=13 biological replicates, myocarditis n=11 biological replicates). Positive values indicate higher values with combined lysis and sonication compared to EV isolation. The solid line indicates mean bias (6.0 ng/L); dotted lines indicate 95% limits of agreement (−11 to 23 ng/L). **(C)** Passing-Bablok regression comparing EV cTnT/plasma cTnT ratios using combined lysis and sonication vs. conventional EV isolation (n=24 biological replicates; NSTEMI n=13 biological replicates, myocarditis n=11 biological replicates). The solid line represents the regression fit (intercept: 0.031, 95% CI: −0.010 to 0.058; slope: 0.881, 95% CI: 0.608 to 1.536); the dashed line represents the identity line. **(D)** Bland-Altman plot comparing EV cTnT/plasma cTnT ratios using combined lysis and sonication vs. conventional EV isolation (n=24 biological replicates; NSTEMI n=13 biological replicates, myocarditis n=11 biological replicates). Positive values indicate higher ratios with combined lysis and sonication compared to EV isolation. The solid line indicates mean bias (0.023); dotted lines indicate 95% limits of agreement (−0.096 to 0.14).

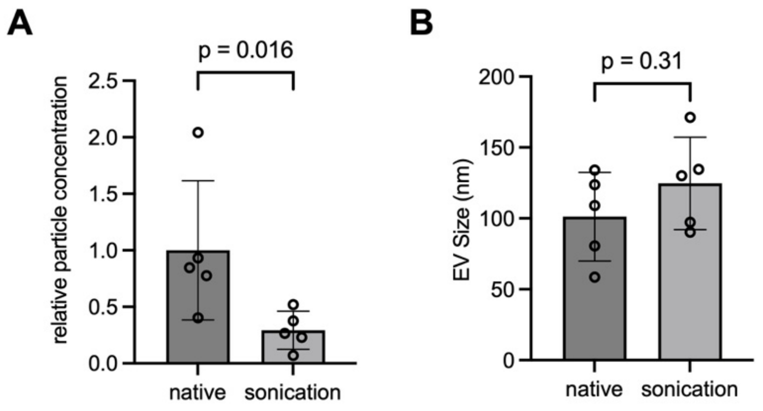

Supplemental Figure S3. Nanoparticle tracking analysis before and after combined lysis and sonication. EVs were isolated from plasma of healthy controls before and after combined lysis and sonication using an EV isolation kit. **(A)** Relative particle concentration in native vs. processed (sonication) samples. Data are presented as mean ± SD, Mann-Whitney U test, n=5 biological replicates with three technical replicates each. **(B)** EV size (nm) of corresponding native and processed (sonication) samples. Data are presented as mean ± SD, Mann-Whitney U test, n=5 biological replicates with three technical replicates each.

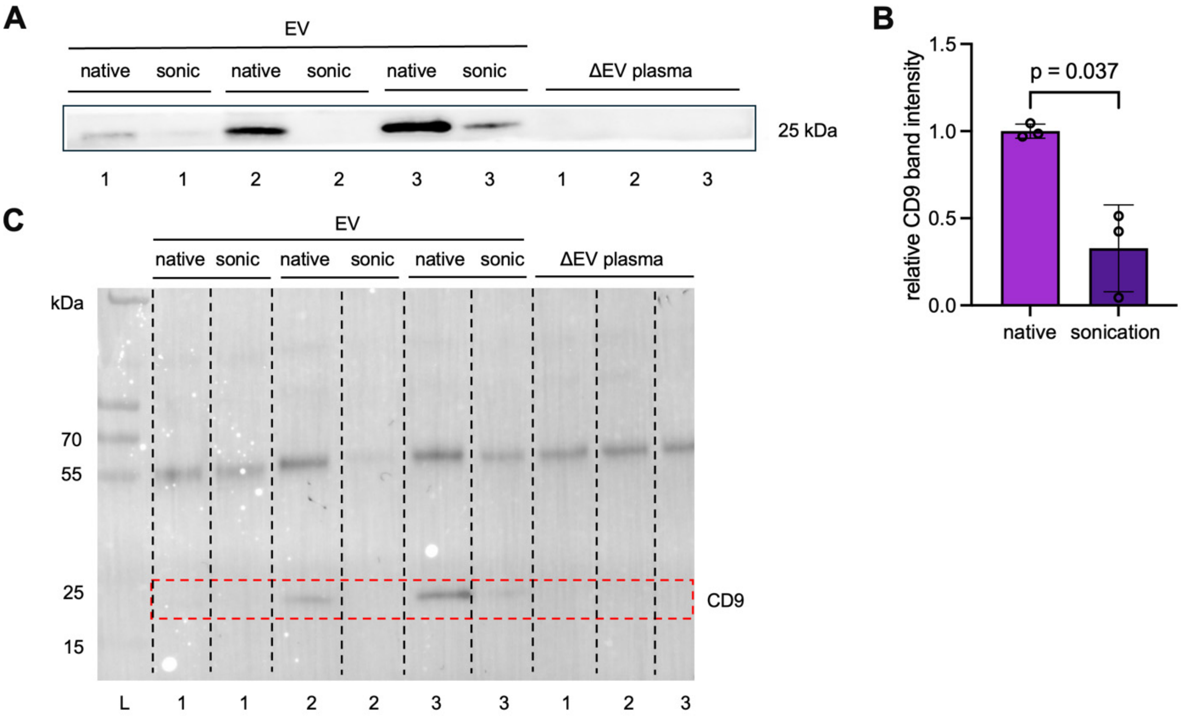

Supplemental Figure S4. Immunoblotting for EV marker CD9 in EV isolates and EV-depleted plasma of healthy controls. (A) Immunoblotting for CD9 in native and processed (sonic) EV isolates (EV) from three biological replicates (1-3), and EV-depleted plasma (ΔEV plasma, replicates 1-3). (B) Quantification of relative CD9 band intensity in native and processed (sonication) EV samples, normalized to native control. Data presented as mean ± SD, paired t-test, n=3 biological replicates. (C) Full immunoblot of Figure S4A showing specific enrichment of CD9 in native and processed EV isolates, and absence of enrichment in EV-depleted plasma. L=ladder.

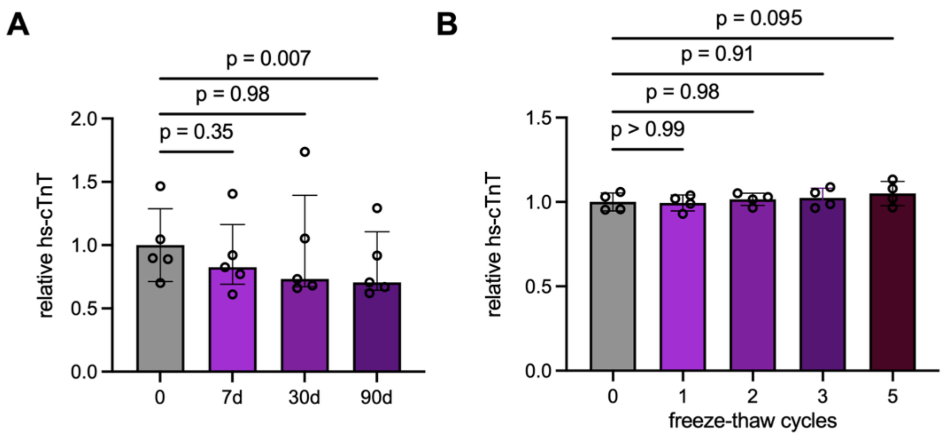

Supplemental Figure S5. Storage and freeze-thaw stability of EV-bound cTnT. (A) Citrated plasma of healthy controls was stored at −80 °C. EV were isolated by ultracentrifugation, and EV-bound hs-cTnT was measured immediately after collection and at 7, 30, and 90 days after initial freezing, normalized to the mean at baseline. Data are presented as mean ± SD, Shapiro-Wilk normality test followed by repeated-measures one-way ANOVA with Geisser-Greenhouse correction and Dunnett's post-hoc test, n=5 biological replicates. **(B)** Freeze-thaw stability of EV-bound hs-cTnT after 0, 1, 2, 3, and 5 cycles in plasma from healthy controls. For each cycle, separate aliquots were frozen at −80 °C for at least 2h and thawed on ice for 1h, followed by combined lysis and sonication, and normalized to the mean at baseline. Data are presented as mean ± SD, Shapiro-Wilk normality test followed by repeated-measures one-way ANOVA with Geisser-Greenhouse correction and Dunnett's post-hoc test, n=4 biological replicates.
